# Ninoa *T. cruzi* Strain Modifies the Expression of microRNAs in Cardiac Tissue and Plasma During Chagas Disease Infection

**DOI:** 10.3390/pathogens13121127

**Published:** 2024-12-20

**Authors:** Rogelio F. Jiménez-Ortega, Ricardo Alejandre-Aguilar, Nancy Rivas, Fausto Sánchez, Fausto Sánchez-Muñoz, Martha A. Ballinas-Verdugo

**Affiliations:** 1 Laboratorio de Genómica del Metabolismo Óseo, Instituto Nacional de Medicina Genómica (INMEGEN), Mexico City 14610, Mexico; rogeliofrank.jimenez@uneve.edu.mx; 2 Unidad de Acupuntura Rehabilitatoria, Universidad Estatal del Valle de Ecatepec (UNEVE), Ecatepec 55210, Estado de México, Mexico; 3 Departamento de Parasitología, Escuela Nacional de Ciencias Biológicas (ENCB), Instituto Politécnico Nacional (IPN), Mexico City 07738, Mexico; rialejandre@yahoo.com.mx (R.A.-A.); caliope_rivas@yahoo.com.mx (N.R.); 4 División de Ciencias Biológicas y de la Salud, Universidad Autónoma Metropolitana Xochimilco (UAM-X), Mexico City 04960, Mexico; sygf2122@correo.xoc.uam.mx; 5 Departamento de Fisiología, Instituto Nacional de Cardiología Ignacio Chávez (INCICH), Mexico City 14080, Mexico; fausto22@yahoo.com; 6 Departamento de Inmunología, Instituto Nacional de Cardiología Ignacio Chávez (INCICH), Mexico City 14080, Mexico

**Keywords:** Chagas disease, *Trypanosoma cruzi*, miRNAs, biomarkers, extracellular vesicles, chronic chagasic cardiomyopathy

## Abstract

**Background:** Chronic chagasic cardiomyopathy is the most severe clinical manifestation of Chagas disease, which affects approximately seven million people worldwide. Latin American countries bear the highest burden, with the greatest morbidity and mortality rates. Currently, diagnostic methods do not provide information on the risk of progression to severe stages of the disease. Recently, microRNAs (miRNAs) have been proposed as promising tools for monitoring the progression of Chagas disease. This study aimed to analyze the expression profiles of the miRNAs miR-1, miR-16, miR-208, and miR-208b in cardiac tissue, plasma, and plasma extracellular vesicles from Ninoa TcI-infected mice during the acute and indeterminate phases of Chagas disease. **Methods**: The cardiac-specific miRNAs and miR-16 levels were examined in all samples using RT-qPCR. Additionally, pathway analysis was performed to investigate the impact of potential miRNA target genes across various databases. **Results**: Elevated miR-208b expression was observed in cardiac tissue and plasma during the acute phase. Bioinformatic analysis identified three pathways implicated in disease progression: phosphatidylinositol 3-kinase signaling, Fc gamma receptor-mediated phagocytosis, and leukocyte transendothelial migration, as well as cholinergic synapse pathways. **Conclusions**: MiR-208b was upregulated during the acute phase and downregulated in the indeterminate phase, suggesting it may play a crucial role in disease progression.

## 1. Introduction

The heart is a vital organ and central to the cardiovascular system. Unfortunately, it is susceptible to various pathologies, including cardiomyopathies and cardiovascular diseases [1]. Cardiomyopathies are characterized by structural and functional abnormalities in the myocardium and encompass a heterogeneous group of conditions, such as arrhythmias, progressive heart failure, and stroke. Currently, the four main subtypes of cardiomyopathies are restrictive cardiomyopathy, hypertrophic cardiomyopathy, arrhythmogenic ventricular cardiomyopathy, and dilated cardiomyopathy (DCM) [2,3,4,5].

Heart failure, often a consequence of DCM, can be caused by infectious agents, including viruses (e.g., coxsackievirus B2, herpes virus, Epstein–Barr virus, parvovirus B19, and adenoviruses), bacteria (e.g., *Borrelia* spp.), and the protozoan *Trypanosoma cruzi* (*T. cruzi*) [2,3,6].

*T. cruzi* is the causative agent of Chagas disease, a kinetoplastid protozoan parasite transmitted through the urine and feces of infected, blood-feeding triatomine bugs. It has a complex life cycle, alternating between a proliferative intracellular form in mammals and a proliferative form in the insect vector, which differ morphologically and functionally [7]. Approximately 75 million people live in areas at risk of contracting Chagas disease. This illness is potentially fatal, primarily due to its cardiac and digestive complications. Chronic chagasic cardiomyopathy (CCC) is associated with parasite persistence, fibrosis, inflammation, autoimmunity, dysautonomia, right bundle branch block, left anterior fascicular block, rhythm disturbances, intraventricular obstructions, and ischemic abnormalities [8,9,10,11].

Predicting disease progression to the chronic phase in infected individuals remains challenging. However, microRNAs (miRNAs)—small (~22 nucleotides), endogenous, non-coding RNAs—have emerged as crucial post-transcriptional regulators of gene expression. They play key roles in various biological processes across humans, animals, plants, and other organisms. Notably, due to their stability and small size, miRNAs can be detected in biological fluids, such as blood, plasma, serum, saliva, and urine. In addition, circulating miRNAs have been recognized as potential biomarkers for infectious and non-infectious diseases. Additionally, extensive research has demonstrated that miRNAs contribute to cellular communication via extracellular vesicles (EVs) [12,13,14].

Several studies have investigated miRNAs in the context of Chagas disease. For instance, Rodrigues et al. (2014) found that miR-1, miR-133a, miR-133b, miR-208a, and miR-208b were significantly altered in CCC [15]. Similarly, Linhares et al. (2018) reported elevated miR-208a levels during the chronic indeterminate phase, suggesting it could be a biomarker for predicting Chagas disease progression [16]. EVs have also been extensively characterized using techniques such as transmission electron microscopy, nanoparticle tracking analysis, and 2D LC−MS/MS. Bayer-Santos et al. (2013) analyzed three fractions from the epimastigote and metacyclic forms of *T. cruzi*, identifying 345 proteins in the epimastigote stage, 265 in the metacyclic stage, and 243 common to both forms [17]. Regarding miRNAs, high levels of miR-21 and miR-146a have been detected in plasma EVs from Ninoa *T. cruzi*-infected mice [18].

Given this background, our objective was to explore the expression levels of miR-1, miR-208, miR-208b, and miR-16 in cardiac tissue, plasma, and plasma EVs during the acute and indeterminate phases in Ninoa TcI-infected mice.

## 2. Materials and Methods

### 2.1. Parasites and Mice

Ninoa (MHOM/MX/1986/Ninoa) TcI strains were used, originally isolated from an acute human case of Chagas disease [19]. This strain was maintained in *Meccus pallidipenis*. To propagate the strain, fecal samples from infected triatomines were used to inoculate CD1 mice intraperitoneally. At the peak of parasitemia (27 days post-inoculation), 30 first-instar *M. pallidipennis* nymphs were fed on infected mice. This process was repeated to maintain the strain. Metacyclic trypomastigotes were subsequently obtained from the urine and feces of infected triatomines. Forty-six CD1 mice (9–12 weeks old, weighing 25 ± 2 g) were housed under standard conditions with *ad libitum* access to food and water at room temperature and a 12/12 h light/dark cycle.

### 2.2. Experimental Infections by T. cruzi

This case–control study involved histological and molecular analysis. Twenty-six mice were infected intraperitoneally with 1000 metacyclic trypomastigotes. Six mice were used for histopathological evaluation, while twenty were used for miRNA expression analysis (considered cases). Another group of twenty mice received parasite-free urine/feces through the same route and served as controls. All animal procedures adhered to the ethical standards established in the Declaration of Helsinki and comparable guidelines. The study was reviewed and approved by the Ethics Committee of the Instituto Nacional de Cardiología Ignacio Chávez (approval number: 16-964).

### 2.3. Sample Collection

Twenty mice were sacrificed 21 days post-infection to analyze the acute phase: 10 infected (cases) and 10 controls. Another group was sacrificed 81 days post-infection to study the indeterminate phase, characterized by an absence or undetectable level of circulating parasites. Previous parasitemia curve evaluations indicated that the acute phase ends around 60 days post-inoculation, after which parasites become undetectable in blood [18]. Therefore, 80 days post-inoculation was considered the start of the indeterminate phase. All mice were euthanized using an overdose of sodium pentobarbital (100–150 mg/kg I.P.). Blood samples were obtained via cardiac puncture, and hearts were removed for analysis.

### 2.4. Histological Study of Ninoa TcI-Infected Mice

To assess pathogenesis, hearts from six infected mice were fixed in a 10% buffered formalin solution, embedded in paraffin, sectioned at 5 μm, deparaffinized, and stained with hematoxylin–eosin. The sections were examined at 40× magnification to evaluate parasitism and inflammation.

### 2.5. Isolation of Total RNA Enriched for miRNAs from Heart, Plasma, and Plasma EVs

Samples from the heart apex, plasma, and plasma EVs of Ninoa TcI-infected mice were processed for miRNA extraction. Total RNA was extracted using QIAzol^®^ Lysis Reagent (QIAGEN, Hilden, Germany): 500 μL for plasma and 700 μL for tissue and EVs. During RNA purification, 3.5 μL (QIAGEN, Hilden, Germany), cel-miR-39 Spike-In Control^®^ (1.6 × 108 copies/μL) was added to plasma and EVs samples. RNA was purified using the miRNeasy^®^ Kit (QIAGEN, ID: 217004, Hilden, Germany), miRNeasy^®^ Serum/Plasma Kit (QIAGEN, ID: 217184, Hilden, Germany), and the exoRNeasy^®^ Serum/Plasma Midi Kit (QIAGEN, Hilden, Germany), following the manufacturer’s protocol.

### 2.6. Identification of Cardiac miRNAs by RT-qPCR

Cardiac-specific miRNAs and miR-16 were selected for their relevance to heart biology, regeneration, and disease. A literature search was focused on Chagas disease, cardiovascular disease, and *Trypanosoma cruzi* using the public database PubMed (https://pubmed.ncbi.nlm.nih.gov/, accessed on 9 April 2023).

cDNA synthesis was performed using the TaqMan^®^/microRNA isolation kit (Applied Biosystems, Foster City, CA, USA), optimized according to the manufacturer’s instructions. For plasma and EVs, 1 µL of total RNA enriched for miRNAs was used directly, while RNA from cardiac tissue was adjusted to a concentration of 25 ng/µL. Specific primers for miR-1 assay (assay ID: 002222), miR-16 (assay ID: 000391), miR-208 (assay ID: 000511), and miR-208b (assay ID: 002290), cel-miR-39 (assay ID: 000200) and U6 (assay ID: 001973) were used in the final 12 µL reaction mix (Supplementary Table S1).

### 2.7. Bioinformatic Analysis

#### 2.7.1. Target Gene Prediction for Each miRNA

To understand the possible role of miRNAs induced by *T. cruzi* infection, a search and analysis of their potential target genes was carried out. Potential target genes were identified if they appeared in at least three databases: TargetScan (http://www.targetscan.org/vert_80/, accessed on 18 April 2023), PicTar (https://pictar.mdc-berlin.de/, accessed on 18 April 2023), miRWalk (http://mirwalk.umm.uni-heidelberg.de/, accessed on 18 April 2023), and miRDB (http://mirdb.org/miRDB/, accessed on 18 April 2023).

#### 2.7.2. Expression Microarray Analysis

Microarray data in the CEL format from the Mouse Genome 430A 2.0 Affymetrix platform were obtained from the Gene Expression Omnibus (GEO) database (accession number: GSE41089) (https://www.ncbi.nlm.nih.gov/geo/, accessed on 18 April 2023). This dataset includes differential gene expression profiles of cardiac tissue from *T. cruzi*-infected and control mice [20].

#### 2.7.3. Data Processing and Differentially Expressed Genes

CEL files were processed using the Robust Microarray Analysis (RMA) method with the Affy package in R software v4.4.2. Fluorescence probe values were converted to numerical data, background-corrected, and normalized using the quantile method. Genes with fold-change values < −1.0 or >1.0 and a false discovery rate (FDR) < 0.05 were considered differentially expressed.

#### 2.7.4. Selection of Potential Candidate Genes

A Venn diagram analysis compared predicted miRNA targets with differentially expressed genes to identify typical candidates. These genes were analyzed using the ShinyGO v0.741 (http://bioinformatics.sdstate.edu/go74/, accessed on 18 April 2023) for gene ontology enrichment analysis and pathway classification. Relevant signaling pathways were identified via STRING-KEGG Pathway (https://string-db.org/, accessed on 18 April 2023), and interaction networks between miRNAs and target genes were constructed using Cytoscape v3.9.1.

### 2.8. Statistical Analysis

Statistical differences between groups in RT-qPCR data were assessed using the unpaired Student’s *t*-test or Mann–Whitney U test (*p* < 0.05), using Graph Pad Prism version 6.0. Data are presented as means ± standard error. Normality and variance assumptions were tested prior to analysis.

## 3. Results

### 3.1. Histopathology Study

**Figure 1:**
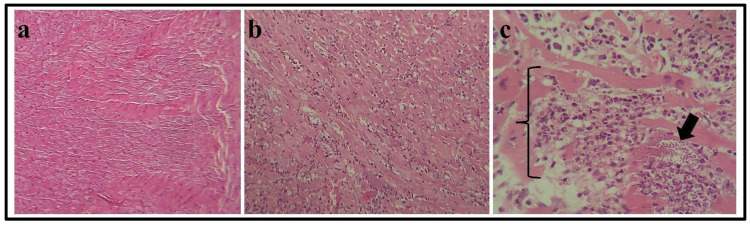
Infection and histology. (**a**) Histological section of regular mouse cardiac tissue. (**b**) Histological section of cardiac tissue during the acute phase. The presence of mature lymphocytes derived from amastigote nests is observed. (**c**) Histological section of cardiac tissue during the indeterminate phase. The arrow indicates amastigotes nests, and the square brackets highlight the inflammatory process.

Histological analysis reveals significant differences between the cardiac tissue of infected mice and controls. In the control group, the cardiac muscle showed normal morphology with no observable alterations (Figure 1a). In contrast, mice infected with the Ninoa strain exhibited myocarditis with severe damage to the myocardium, characterized by numerous amastigote nests with irregular edges (Figure 1b). Additionally, the interstitial spaces of the myocardium showed abundant chronic inflammatory infiltration composed mainly of mature lymphocytes. Extensive lysis of cardiomyocytes was evident, highlighting the severity of the infection (Figure 1c).

### 3.2. Cardiac-Specific miRNAs and miR-16 Expression in Ninoa TcI-Infected Mice

**Figure 2:**
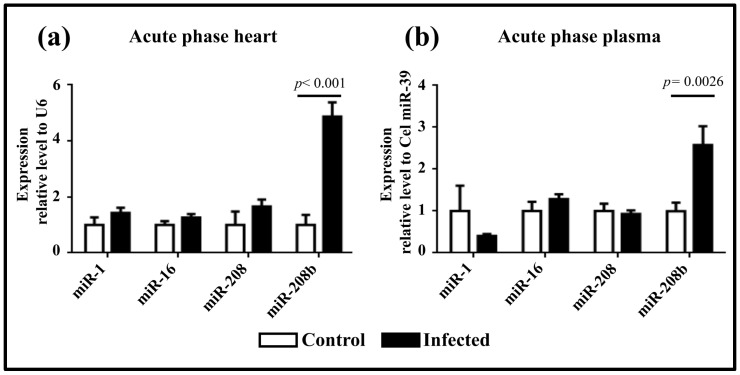
miRNA expression levels during the acute phase. (**a**) Expression levels in cardiac tissue infected with the Ninoa strain. (**b**) Expression levels in plasma from mice infected with the Ninoa strain. Cardiac tissue samples were normalized to U6, and plasma samples were normalized to cel-miR-39.

RT-qPCR analysis assessed the expression of three cardiac-specific miRNAs and miR-16. During the acute phase, the expression level of miR-208b was significantly elevated in both heart tissue (4.87 ± 0.51 R.U.) (Figure 2a) and plasma (2.58 ± 0.45 R.U.) (Figure 2b) of infected mice compared to controls. However, no statistically significant differences were observed for miR-1, miR-16, and miR-208.

**Figure 3:**
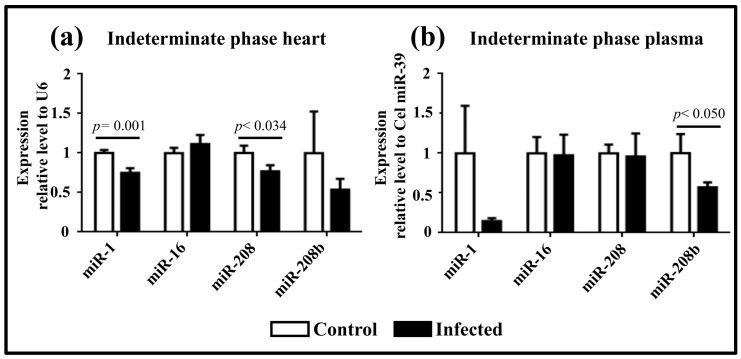
miRNA expression levels during the indeterminate phase. (**a**) Expression levels in cardiac tissue infected with the Ninoa strain. (**b**) Expression levels in plasma from mice infected with the Ninoa strain. Cardiac tissue samples were normalized to U6, and plasma samples were normalized to cel-miR-39.

In the indeterminate phase, miR-1 (0.75 ± 0.06 R.U.) and miR-208 (0.77 ± 0.08 R.U.) showed a notable downregulation in heart tissue compared to controls (Figure 3a), indicating dynamic changes in miRNA expression during infection. In plasma, miR-208b expression was downregulated (0.57 ± 0.06) in infected mice compared to controls (Figure 3b).

### 3.3. Expression of miRNAs in Plasma EVs from Ninoa TcI-Infected Mice

**Figure 4:**
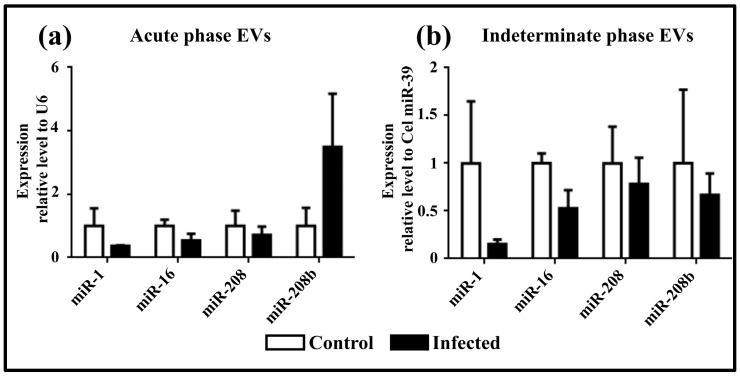
miRNA expression levels in EVs. (**a**) Expression levels in plasma from mice infected with the Ninoa strain during the acute phase. (**b**) Expression levels in plasma from mice infected with the Ninoa strain during the indeterminate phase. Cardiac tissue samples were normalized to U6, and plasma samples were normalized to cel-miR-39.

The expression levels of four cardiac miRNAs were also analyzed in EVs isolated from the plasma of Ninoa TcI-infected mice during both the acute and indeterminate phases of Chagas disease. During the acute phase, miR-1, miR-16, and miR-208 showed a downward trend, while miR-208b showed an upward trend. However, these changes were not statistically significant (Figure 4a). All miRNAs showed a downward trend in the indeterminate phase, but no statistically significant differences were observed (Figure 4b).

### 3.4. Prediction of miRNA Target Genes Associated with T. cruzi

**Figure 5:**
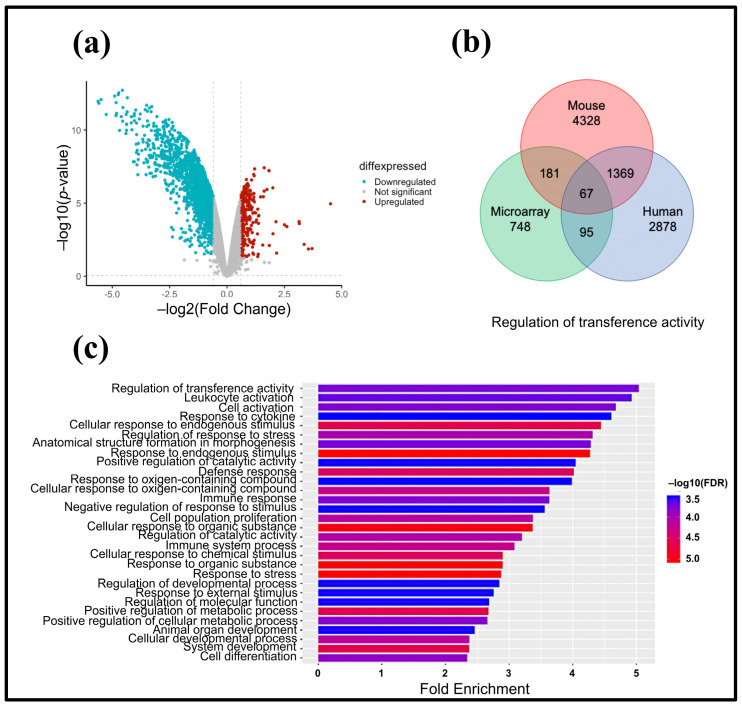
Bioinformatic analysis (**a**) Volcano plot showing underexpressed and overexpressed genes, with a fold-change less than −1 or greater than 1 and a *p*-value < 0.05. (**b**) Venn diagram illustrating the overlap of putative target genes for miR-1, miR-16, miR-208, and miR-208b from humans and mice, along with differentially expressed genes from Mouse Genome 430A 2.0 microarrays from the Affymetrix platform. (**c**) Enrichment pathways identified using ShinyGO v0.741 software and the number of participating genes in each pathway.

Predicted target genes for miR-1, miR-16, miR-208, and miR-208b were identified based on consistency across at least three databases. For human miRNAs, 698 target genes were identified for miR-1, 3917 for miR-16, 110 for miR-208, and 101 for miR-208b. For mouse miRNAs, 2867 target genes were identified for miR-1, 3905 for miR-16, 108 for miR-208, and 78 for miR-208b. These potential target genes were cross-referenced with the downregulated genes identified in the microarray differential expression analysis based on the criterion that high miRNA levels correspond to low target gene expression. Genes with a fold change (FC) of ≤−1.0 were considered downregulated (Figure 5a). This approach identified 67 putative dysregulated target genes for the analyzed miRNAs. The interaction between predicted target genes from human, mouse, and microarray data is shown in Figure 5b.

### 3.5. Analysis of Interaction Networks

Gene ontology (GO) enrichment analysis of the 67 target genes was performed using ShinyGO v0.741, revealing 30 biological processes with significant false discovery rate (FDR) values (Figure 5c). Subsequently, canonical pathway analysis identified 41 KEGG signaling pathways, 15 of which were associated with Chagas disease and *T. cruzi* infection.

**Figure 6:**
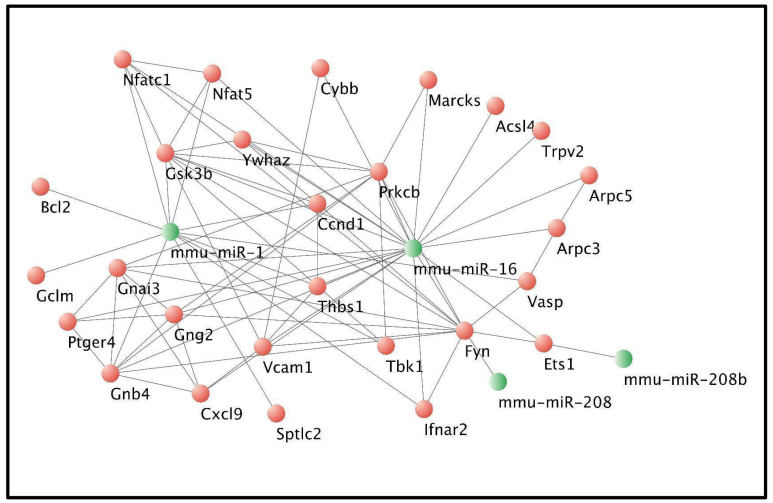
miRNA-Target gene interaction network. Interaction network of miR-1, miR-16, miR-208, and miR-208b with 26 potential target genes involved in enriched signaling pathways. These genes are associated with inflammatory processes and *T. cruzi* virulence. Key genes include Nuclear Factor of Activated T cells 1 (NFATc1), Nuclear Factor of Activated T cells 5 (NFAT5), cAMP Responsive Element Binding Protein 1 (CREB1), B-cell lymphoma 2 (BCL2), and Glycogen Synthase Kinase 3 beta (GSK3B).

The identified genes involved in these pathways were used to construct an interaction network between miRNAs and their respective target genes using Cytoscape. Notably, genes such as *Bcl2*, *Gsk3b*, *Nfat5*, and *Nfatc1* were integrated into this network. Previously identified as putative targets of miR-146a, these genes are implicated in the pathogenesis induced by *T. cruzi* infection (Figure 6) [18].

## 4. Discussion

To evaluate the role of miRNAs in gene regulation during CCC, we performed an integrative analysis of miRNA and mRNA expression in the mouse genome. We examined the expression of miR-1, miR-16, miR-208, and miR-208b in cardiac tissue, plasma, and EVs from Ninoa TcI-infected mice during the acute and indeterminate phases of Chagas disease.

*Trypanosoma cruzi* has a complex life cycle with numerous strains and clones. The current international consensus classifies *T. cruzi* into seven discrete typing units (TlcI–TlcVI and Tcbat) [21,22,23], with TcI being the predominant strain in Mexico and Guatemala [24]. Also, other studies have mentioned the predominance of lineage TcI in triatomines, humans, and murine infection [25,26,27,28,29].

Our findings with the cardiotropic Ninoa TcI strain show significant amastigote nests and inflammatory infiltrates, with myocarditis evident in infected cardiac tissue compared to healthy controls. Our results are similar to those of Espinoza et al.’s (2010) study [30].

Recent studies have focused on understanding miRNA roles in Chagas disease progression. Ferreira et al. (2015) reported the deregulation of miR-1, miR-133a, miR-133b, miR-208, and miR-208b in CCC [15]. Notably, miR-208b was overexpressed in cardiac tissue and plasma during the acute phase, while during the indeterminate phase, miR-208b was underexpressed in cardiac tissue but overexpressed in plasma [25]. Our results align with these findings, except for miR-208b plasma expression in the indeterminate phase, which remained low in our study.

Linhares et al. (2018) correlated circulating miRNA expression profiles in plasma with CCC severity. They observed increased miR-208a expression in humans during the indeterminate phase, suggesting its potential role in TGF-β-induced fibrosis and hypertrophy. They proposed miR-208a as a predictive marker for CCC risk [16]. In our study, miR-208b was the only cardiac-specific miRNA deregulated in both phases of Ninoa TcI infection.

Few studies have investigated cardiac-specific miRNAs as potential biomarkers in fluids like urine or serum during Chagas disease. Ballinas et al. (2021) reported the overexpression of miR-21 and miR-146a, suggesting miR-146a as a candidate biomarker [18].

Cardiac-specific miRNAs, such as miR-208a and miR-208b, play crucial roles in cardiovascular diseases, including hypertrophic and arrhythmogenic cardiomyopathies. Callis et al. (2009) demonstrated that miR-208a overexpression regulates hypertrophic growth and the cardiac conduction system, implicating β-MHC expression and fibrosis [31,32].

In our study, we predicted sixty-seven target genes. We identified *Bcl2*, *Gsk3*, *Nfat5*, and *Nfatc1* as key players in Chagas disease pathogenesis, and our bioinformatics analysis identified 15 signaling pathways related to Chagas disease development. Key pathways include phosphatidylinositol 3-kinase (PI3K), Fc gamma receptor-mediated phagocytosis, leukocyte transendothelial migration, and cholinergic synapse pathways. In the PI3K pathway, *T. cruzi*-derived neurotrophic factors bind to host cell tyrosine kinases, potentially regulating survival, proliferation, and nutrient availability [33]. However, these short-lived interactions may not explain long-term parasitism [34]. Elevated serum levels of miR-19a-3p, miR-29b-3p, and miR-30a-5p correlate with cardiac injury severity during the chronic phase [35].

Our results suggest that miR-1 and miR-16 regulate immune response-related genes, potentially influencing host defense mechanisms to prevent chronic infection. These findings align with Wu et al. (2021), who identified immune-related pathways such as natural killer cell-mediated cytotoxicity, leukocyte transendothelial migration, the chemokine signaling pathway, and the PI3K−Akt signaling pathway [36].

The Fc gamma receptor pathway is crucial for immune functions, including phagocytosis and cytokine production, and may enhance anti-*T-cruzi* responses in infected tissues [37]. Additionally, leukocyte transendothelial migration involves interactions with endothelial cells, contributing to the inflammatory response during infection. For example, the chemokine CCL2 increases *T. cruzi* recruitment in vivo, which suggests a pre-existing inflammation may help regulate subsequent tissue infection [38]. This process is essential for pathogen clearance, but the mechanisms by which T. cruzi crosses the endothelial barrier remain unclear [39,40].

Endothelial cells express miRNAs that regulate leukocyte trafficking, which may control vascular inflammation during Chagas disease [41]. The cholinergic synapse pathway may contribute to symptoms like reduced anal sphincter relaxation, as autonomic nervous system dysfunction is linked to motility disorders such as megacolon in Chagas patients [42].

MiRNAs have emerged as key regulators of complex biological processes in various diseases, such as different types of cancer, altered physiological states, and cardiac diseases. Still, reliable results depend on proper normalization from suitable reference genes. However, despite the increasing number of studies evaluating miRNAs in cardiac diseases, a consensus has yet to be reached on the best reference genes, as they can exhibit high variability and are only reliable under certain conditions. For example, U6 levels can vary between individuals and different populations, and their expression can decrease after the freezing and thawing of serum and plasma. Currently, U6 is one of the most widely used endogenous controls in miRNA research. However, it has been reported that this miRNA can exhibit fluctuations in the serum of patients with nasopharyngeal carcinoma [43] and patients with sepsis or liver fibrosis [44].

Cel-miR-39 is a quantified synthetic miRNA used for RNA extraction and normalization procedures, which directly correlates with the total amount of RNA recovered, so it has been used as a suitable endogenous control for plasma/serum samples. On the other hand, some authors have sought to evaluate reference miRNAs in cardiac tissue, but a consensus has yet to be reached on the best reference genes. In work carried out by Masè et al. (2017), they evaluated the stability of five reference genes (U6, SNORD48, SNORD44, miR-16, and 5S) in human cardiac tissue, with SNORD48 being the best reference gene and U6 being the worst [45]. These variations observed in the U6 and Cel-miR-39 controls may differ between individuals, biological conditions, or diseases, so probably in our study, when working with a murine model, where mice are kept under controlled conditions, no fluctuations were observed in the expression levels of both U6 and Cel-miR-39. Recently, some research groups have reported that the expression of miRNAs in serum and plasma is consistent, so using these miRNAs as an internal reference can be used as a standard control [43].

In conclusion, our findings highlight miR-208b as a key cardiac-specific miRNA deregulated during acute Chagas disease. While EV-associated miRNA changes were not statistically significant, further analysis with individual samples and diverse Mexican strains is essential to understand Chagas disease progression.

## Data Availability

The original contributions presented in the study are included in the article. The data presented in this study are available on request from the corresponding author.

## Supplementary Materials

The following supporting information can be downloaded at: https://www.mdpi.com/article/10.3390/pathogens13121127/s1, Table S1: Sequences of the miRNAs analyzed.
