# Supplementary material for: Ninoa T. cruzi Strain Modifies the Expression of microRNAs in Cardiac Tissue and Plasma During Chagas Disease Infection
Source: Pathogens. 2024 Dec 20;13(12):1127. doi: 10.3390/pathogens13121127 (PMC11679500; doi:10.3390/pathogens13121127)
Supplement: Supplementary file 1 [file pathogens-13-01127-s001.zip › pathogens-3313297-supplementary.pdf]

Supplementary Table S1. Sequences of the miRNAs analyzed.

Assay ID	miRNA	Specie	Sequence
462049_mat	mmu-miR-1	Mouse	ACAUACUUCUUUAUAUGCCCAUA
002489	mmu-miR-16	Mouse	CCAGUAUUGACUGUGCUGCUGA
462036_mat	mmu-miR-208a	Mouse	GAGCUUUUGGCCCGGGUUAUAC
462460_mat	mmu-miR-208b	Mouse	AAGCUUUUUGCUCGCGUUAUGU
NR_004394	U6 snRNA	Mouse	GTGCTCGCTTCGGCAGCACATATACTAAAATTGGAACGATACAGAGAAGATT
478293_mir	cel-miR-39-3p	C. elegans	UCACCGGGUGUAAAUCAGCUUG
